# The Impact of Entrepreneurs’ AI Literacy on Entrepreneurial Resilience: The Role of AI Anxiety and Social Support

**DOI:** 10.3390/bs15121741

**Published:** 2025-12-16

**Authors:** Yirong Liu, Haiqing Hu, Weiwei Kong

**Affiliations:** School of Economics and Management, Xi’an University of Technology, Xi’an 710054, China; 1220511010@stu.xaut.edu.cn (Y.L.); 1210512016@stu.xaut.edu.cn (W.K.)

**Keywords:** AI literacy, AI anxiety, entrepreneurial resilience, social support, conservation of resources theory

## Abstract

In the contemporary entrepreneurial environment increasingly shaped by artificial intelligence, the Artificial intelligence (AI) literacy of entrepreneurs plays an essential role in enhancing entrepreneurial resilience. However, the underlying mechanisms that explain this relationship remain inadequately explored. Grounded in the Conservation of Resources (COR) theory, this study examines the effect of entrepreneurs’ AI literacy on entrepreneurial resilience, specifically assessing the mediating effect of AI anxiety and the moderating effect of social support. Data were obtained from 330 Chinese entrepreneurs using a two-wave survey design. The analysis revealed a significant positive association between AI literacy and entrepreneurial resilience. Furthermore, AI anxiety mediates this relationship, suggesting that AI literacy enhances entrepreneurial resilience by alleviating AI anxiety. Moreover, all three forms of social support were found to buffer the negative effect of AI anxiety on resilience. Notably, the moderating effects of subjective support and support utilization are significantly stronger than those of objective support, highlighting the distinct roles of different support types. These findings deepen theoretical insight into the psychological pathways linking AI literacy to resilience and offer practical guidance for entrepreneurs in the AI era.

## 1. Introduction

The BANI (Brittle, Anxious, Nonlinear, Incomprehensible) framework aptly characterizes the contemporary business landscape, thereby underscoring the unprecedented challenges that entrepreneurial activities face (Evseeva et al., 2021; Demirel & Camcı, 2024). In this context, the core task for entrepreneurs has shifted from merely pursuing growth or innovation to navigating uncertainty, ensuring survival, and sustaining operations. This implies that entrepreneurial resilience, defined as the ability to endure, adapt, and bounce back from adversity, has become a critical ability (Korber & McNaughton, 2018; Shore et al., 2024). It not only motivates entrepreneurs to persevere under difficult conditions but also facilitates the attainment of favorable results like entrepreneurial success and performance improvement during difficult times (Badzaban et al., 2021; Santoro et al., 2020; Mignenan, 2021). Consequently, investigating how entrepreneurs can enhance their resilience within this complex contemporary environment is of paramount importance.

Among the many forces shaping the BANI era, AI stands out as a disruptive technology that is acting as a transformative force in reshaping entrepreneurial activities (Obschonka & Audretsch, 2020; Duong & Nguyen, 2024). To effectively navigate technological advancements and address technical challenges, entrepreneurs must possess commensurate advanced technological literacy and professional competencies (Shatila et al., 2025). AI literacy is conceptualized as an ability that allow individuals to comprehend, utilize, and critically assess artificial intelligence systems and their outcomes in an ethically responsible manner (Long & Magerko, 2020; B. Wang et al., 2023; Imjai et al., 2025). This literacy enables entrepreneurs to effectively embed AI into their entrepreneurial practices, and to leverage the technology to navigate and contribute within complex environments (Ng et al., 2021; Al-Abdullatif & Alsubaie, 2024). The turbulent entrepreneurial context necessitates that entrepreneurs first rely on AI literacy to secure survival, prior to seeking further growth and development. Consequently, compared to examining its value in enabling traditional outcomes, a deeper inquiry into how AI literacy enhances entrepreneurial resilience holds paramount strategic significance.

Scholars have recognized AI as an important technological tool influencing entrepreneurial resilience (Hu et al., 2023; Shore et al., 2024). Yet, the precise pathways by which entrepreneurs’ capacity to navigate AI, namely, their AI literacy, impacts entrepreneurial resilience remain under-explored. Existing research on AI literacy has predominantly focused on the educational sector (Imjai et al., 2025; Wijaya et al., 2024; Shi et al., 2025). Within the entrepreneurship context, scholarly attention has only preliminarily examined its predictive role in shaping entrepreneurial intention (Pham et al., 2025; Duong, 2025; Nguyen et al., 2024). The critical issue of whether entrepreneurs can leverage AI literacy to enhance their actual resilience in coping with adversity remains largely unexamined. Furthermore, some studies suggest that digital literacy can bolster entrepreneurial resilience by enhancing innovation capabilities or revealing technological application potential (Shatila et al., 2025; Santos et al., 2023). However, AI literacy emphasizes interacting with AI that possesses more biological and social attributes (Lee & Jeon, 2025). Whether this concept effectively enhances resilience in high-pressure entrepreneurial environments still lacks empirical evidence. Accordingly, we examine the influence of AI literacy on entrepreneurial resilience to address the aforementioned gap. Our first research question (RQ1) is: Does entrepreneurs’ AI literacy influence their entrepreneurial resilience?

To investigate the underlying mechanisms, this study draws on the COR theory. This theory posits that individuals who possess a more substantial resource reservoir are better at avoiding threats of resource loss and acquiring new resources, which providing an appropriate theoretical lens for this research (Hobfoll et al., 2018). Applying this perspective, AI literacy acts as an essential resource for entrepreneurs, playing a protective role in the AI era (Qiu et al., 2025). Entrepreneurs equipped with advanced AI literacy can more effectively understand and navigate AI technologies, thereby reducing concerns about loss of control over technology and its negative consequences (Schiavo et al., 2024; Pan et al., 2025). This effectively mitigates the stress response triggered by uncontrollability and uncertainty, known as AI anxiety (Cengiz & Peker, 2025; Uygungil-Erdogan et al., 2025). Entrepreneurs are particularly sensitive to resource loss, as it can directly lead to entrepreneurial failure (Lanivich, 2015). Therefore, mitigating their anxiety is of critical importance. A reduction in AI anxiety means that fewer psychological resources are depleted, allowing entrepreneurs to allocate more cognitive and emotional resources towards coping with adversity, thereby enhancing their entrepreneurial resilience. Research on AI anxiety has extensively examined its antecedents, including individual abilities, technological features, and psychological factors (Tarsuslu et al., 2025; Yao & Xi, 2025; B. Kaya & Çelebi, 2025; D. Chen et al., 2024), as well as its consequences, such as technology attitudes, behavioral intentions, and motivational states (F. Kaya et al., 2024; Y. M. Wang et al., 2024; Y. Y. Wang & Wang, 2022). However, the impact of AI anxiety on entrepreneurial resilience and its mediating mechanism between AI literacy and resilience have not yet been fully explored. This leads to our second research question (RQ2): Does AI anxiety mediate the relationship between AI literacy and entrepreneurial resilience?

Furthermore, COR theory asserts that personal resources are not independent, and their accumulation is facilitated or hindered by environmental conditions through resource caravans (Hobfoll et al., 2018). From this perspective, social support is viewed as a social resource reservoir that entrepreneurs can expand and draw upon to cope with environmental demands and achieve goals (S. Chen et al., 2015; Feng et al., 2025). This support is particularly critical for entrepreneurs in stressful environments (Cohen & Wills, 1985). By providing emotional, informational, and instrumental resources, social support can alleviate AI anxiety, buffer the negative impacts of stress, and enhance psychological well-being and success expectancies (Feng et al., 2025; Huang et al., 2024). Existing research has also shown that social support is a key factor in enhancing resilience (S. Chen et al., 2015; Chang & Yarnal, 2018; Al-alawi et al., 2025). Its moderating role has been validated in various entrepreneurship contexts (Al-Alawi et al., 2023; L. Wang et al., 2025). However, the role of social support within the novel, high-pressure context of AI entrepreneurship remains unclear. This leads to our third research question (RQ3): Does social support moderate the impact of AI anxiety on entrepreneurial resilience?

**Figure 1:**
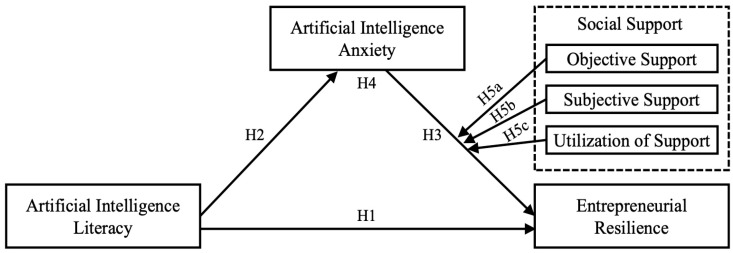
Research model.

In summary, grounded in COR theory, this study investigates the mechanism through which entrepreneurs’ AI literacy influences entrepreneurial resilience, with a specific focus on the mediating role of AI anxiety. It further explores the role of social support, focusing on its moderating effect on the connection between AI anxiety and entrepreneurial resilience. By systematically elucidating these pathways, this research offers valuable insights to advance theoretical understanding and practical guidance for fostering entrepreneurial resilience in the AI-driven context. The detailed research model is presented in Figure 1.

## 2. Research Hypothesis

### 2.1. AI Literacy and Entrepreneurial Resilience

According to COR theory, individuals strive to acquire, retain, and protect resources (Hobfoll, 1989). Resource gain leads to positive outcomes, while resource depletion or loss induce stress (Y. Liu et al., 2024). Within our theoretical framework, AI literacy is positioned as a critical resource essential for entrepreneurs who are adapting to technological change. It drives the enhancement of entrepreneurial resilience through a dual mechanism: proactive resource gain and defensive resource preservation.

On the one hand, entrepreneurs with high AI literacy can continuously acquire cutting-edge AI knowledge and skills. They can proficiently deploy AI tools to identify and exploit innovative applications of AI and potential business opportunities (Duong, 2025), thereby increasing their resource surplus. This capacity allows them to navigate complex technological environments, enhancing adaptability and decision-making to rapidly formulate innovative solutions in crises, thereby bolstering entrepreneurial resilience (Shatila et al., 2025). On the other hand, high AI literacy, by enhancing technological proficiency and operational confidence, effectively reduces the burden of repetitive tasks, optimizes decision-making processes, and lowers the cognitive stress associated with technological uncertainty (Sun et al., 2025). When entrepreneurs perceive that they can cope with external challenges and can leverage AI to allocate resources more efficiently and ensure process stability, their ability to recover from adversity is strengthened (de Esteban Curiel et al., 2024).

Based on this reasoning, we propose the following hypothesis:

**H1.**  *Entrepreneurial AI literacy is positively related to entrepreneurial resilience.*

### 2.2. The Mediating Role of AI Anxiety

COR theory indicates that individuals experience stress reactions and emotional distress when they perceive a threat to, loss of, or failure to gain expected returns on their valued resources, and that possessing resources to cope with such threats is key to reducing these stress reactions (Hobfoll, 1989; Hobfoll, 2001). In the entrepreneurial context of this study, AI anxiety is precisely the stress reaction experienced by entrepreneurs when facing AI-driven technological change. As a critical resource, AI literacy can effectively inhibit AI anxiety by attenuating threat perceptions and protecting self-worth.

On the one hand, high AI literacy facilitates a deeper grasp and judicious use of AI-relevant knowledge and systems, thereby increasing entrepreneurs’ sense of control over the technology (Qiu et al., 2025; Schiavo et al., 2024). This helps dispel fears of AI technology, reduces concerns about skill obsolescence and loss of control, and subsequently alleviates AI anxiety (Zhang & Tong, 2025; Pan et al., 2025; Cengiz & Peker, 2025). On the other hand, AI literacy enables entrepreneurs to clearly define the capability limitations of AI, recognizing that human abilities remain irreplaceable in key areas such as emotional interaction and creative imagination (Komal, 2014). This clear awareness helps entrepreneurs more objectively assess their own irreplaceable self-worth, diminishes excessive worry about being replaced by AI, and thus mitigates anxiety at its source.

Therefore, we propose the following hypothesis:

**H2.**  *Entrepreneurial AI literacy has a significant negative impact on AI anxiety.*

Furthermore, within the COR theoretical framework, AI anxiety, as a stress reaction, constitutes a continuous resource threat that constantly depletes entrepreneurs’ cognitive and emotional reserves. By increasing cognitive load and interfering with cognitive judgment, this stress thereby significantly undermines entrepreneurial resilience.

On one hand, AI anxiety triggers persistent concerns about technological risks and threats, which consumes valuable attentional resources and diverts entrepreneurs’ focus away from their core tasks (Pan et al., 2025; Plass & Kalyuga, 2019). This significantly increases cognitive load and decision fatigue, which can lead to a tendency to avoid challenges. Consequently, it becomes difficult for entrepreneurs to maintain strategic focus and action effectiveness in adversity, ultimately undermining their resilience (Herchenröder et al., 2025). On the other hand, AI anxiety interferes with cognitive judgment, amplifying entrepreneurs’ negative appraisals of technological risks and their own capabilities. Which in turn inhibits their willingness to identify business opportunities, adjust strategies, and persist in their efforts, and weakens their confidence and perseverance in coping with risks. Research clearly indicates that entrepreneurs who are confident and persistent are more likely to transform setbacks into opportunities to enhance entrepreneurial resilience (Guerrero & Walsh, 2024).

Therefore, we propose the following hypothesis:

**H3.**  *Entrepreneurial AI anxiety has a significant negative impact on entrepreneurial resilience.*

In summary, applying the COR theory, this study concludes that AI literacy significantly reduces AI anxiety by functioning as a key personal resource that diminishes threat perceptions and preserves self-worth. Meanwhile, AI anxiety, as a persistent stress reaction, significantly weakens entrepreneurial resilience by depleting cognitive resources and interfering with decision-making. Taken together, these mechanisms suggest that AI anxiety functions as a core mediating mechanism through which AI literacy influences entrepreneurial resilience. That is, AI literacy relieves AI anxiety, thereby conserving and expanding psychological resources, which provides a critical foundation for enhancing entrepreneurial resilience. Based on the foregoing discussion, the following hypothesis is advanced:

**H4.**  *AI anxiety mediates the relationship between AI literacy and entrepreneurial resilience.*

### 2.3. The Moderating Role of Social Support

Furthermore, COR theory also posits that resources tend to cluster, create, and maintain one another within supportive environments (Hobfoll et al., 2018; S. Chen et al., 2015). In this study, social support is conceptualized as a critical social resource. It helps to construct an environment oriented toward resource gain. This environment enables them to access previously unavailable social network resources, clarify their action goals, obtain guidance for success, and integrate into social units that share developmental opportunities (Manix & Abston, 2025; S. Chen et al., 2015). To more precisely reveal how social support moderates the influence of AI anxiety on entrepreneurial resilience, we draw on existing literature to operationalize it as comprising three dimensions: objective support, subjective support, and utilization of support (Xiao, 1994; L. Liu et al., 2025). Objective support refers to the actual tangible aid, financial support, and informational advice received. Subjective support emphasizes emotional-level interactions, manifested as the understanding, acceptance, and validation from family, friends, or partners. Utilization of support reflects an individual’s actual use of social support.

First, high objective support can provide entrepreneurs with solutions to relevant problems, which effectively mitigates their stress (Huang et al., 2024). This assistance helps them to keenly perceive opportunities and persist in action amidst adversity, thereby enhancing resilience (Guerrero & Walsh, 2024). Second, when entrepreneurs fall into self-doubt and fear due to AI anxiety, high subjective support provides Emotional comfort and value affirmation. This, in turn, reduces psychological distress and adverse emotions, facilitates the development of security and belonging, which subsequently enhances their self-efficacy for overcoming challenges (Feng et al., 2025; Manix & Abston, 2025; Ledi, 2024). This process not only conserves the individual’s psychological energy, preventing its depletion under stress, but also enhances their capacity to resist pressure (Zhou et al., 2024). Finally, entrepreneurs with high support utilization are more inclined to proactively communicate and express their needs, enabling them to acquire resources more efficiently and compensate for the psychological resource depletion caused by AI anxiety. This behavioral endowments of actively seeking and utilizing support is critical for entrepreneurs to overcome adversity, allowing them to demonstrate positive adaptability (Hartmann et al., 2022).

In contrast, entrepreneurs with low social support struggle to obtain emotional consolation and instrumental aid. Even when support networks are available, they may fail to access these resources due to an unwillingness to show vulnerability or a lack of skill in seeking help. This situation ultimately exacerbates the negative impact of AI anxiety on their entrepreneurial resilience. These suggest that the higher the level of social support, the weaker the negative impact of AI anxiety on entrepreneurial resilience. Conversely, the lower the level of social support, the stronger this negative impact becomes. Therefore, we propose the following hypotheses:

**H5.**  *Social support moderates the effect of AI anxiety on entrepreneurial resilience.*

**H5a.**  *Objective support moderates the effect of AI anxiety on entrepreneurial resilience.*

**H5b.**  *Subjective support moderates the effect of AI anxiety on entrepreneurial resilience.*

**H5c.**  *Utilization of support moderates the effect of AI anxiety on entrepreneurial resilience.*

## 3. Materials and Methods

### 3.1. Procedure and Participants

This study selects Chinese entrepreneurs as its research sample because they operate in a highly complex BANI world. Their business environment subject to persistent Sino-US trade frictions, intensifying involution-style competition in the domestic market, and the rapid iteration of AI technology. Against this backdrop of high uncertainty and severe resource constraints, investigating how AI literacy helps entrepreneurs enhance resilience and break through growth bottlenecks possesses both significant theoretical value and practical urgency.

With the assistance of directors from entrepreneurial incubators and entrepreneurship parks in four major cities (Beijing, Nanjing, Chongqing, and Xi’an), the research team recruited participants using a convenience sampling approach. The four selected cities are located in the northern, eastern, southwestern, and northwestern regions of China, respectively. This selection was made to ensure diversity in the sample in terms of both geographical coverage and levels of economic development. The participants were founders and key decision-makers from Chinese ventures, who lead resource integration and financial management, and capable of identifying and creating business opportunities. A screening item at the questionnaire outset (“Are you a founder or core decision-maker in your company?”) was employed to ensure sample relevance. Only those responding affirmatively proceeded to the full survey. Data collection was conducted using a combination of an online survey platform (Credamo, Beijing, China) and paper questionnaire. To guarantee data validity and uniqueness, each enterprise was restricted to submitting one questionnaire.

To ensure the quality of the survey instrument, we first conducted a pilot study with 25 eligible entrepreneurs. Based on their feedback, the survey items were adjusted and refined for clarity, semantic accuracy, and logical coherence. The questionnaire clearly stated the research purpose, contents, significance, and confidentiality commitment to enhance both the response rate and data validity. To reduce common method bias (CMB), the study employed a time-lagged data collection design. The first wave (Time 1) was conducted in early March 2025, measuring demographic variables, AI literacy, and AI anxiety. In the second wave (Time 2), three months later, we administered the second survey to measure social support and entrepreneurial resilience. The study administered 430 questionnaires, with 407 returned. Following a review of invalid and excessively incomplete data, 330 responses were retained, yielding a final effective response rate of 76.7%.

The sample comprised 69.7% male and 30.3% female participants, with the majority (82.7%) aged between 26 and 45. In terms of educational background, over half of the entrepreneurs (55.7%) held a bachelor’s degree. In terms of enterprise characteristics, the operating time of enterprises is mostly 2–4 years (53.6%) and employed 11–50 staff (44.6%), while industry distribution was relatively even. These demographic statistics indicate that the sample is diverse and representative (Table 1).

### 3.2. Measurement

This study employed or adapted measurement scales from previously validated instruments in the literature to ensure their validity and reliability. To guarantee clarity and cross-cultural consistency, all non-Chinese scales underwent a rigorous process of translation and back-translation. Followed by two PhD candidates familiar with Chinese culture evaluated each item for its applicability, construct equivalence, and expression accuracy, refining the expressions as needed.

AI literacy in this research is measured as a construct consisting of five dimensions. The first four, AI Basics, AI Proficiency, AI Insight, and AI Analysis, were primarily adopted from Imjai et al. (2025). Drawing from the research of Çelebi et al. (2023) and B. Wang et al. (2023), an AI Ethics dimension was added. The adapted scale consists of 5 dimensions and 11 items, effectively assessing respondents’ understanding and application capabilities regarding AI (Cronbach’s *α* = 0.912). A sample item is, “I have a good understanding of various types of AI and grasp the fundamental principles of AI.”

AI anxiety was measured using the 21-item scale developed by Y. Y. Wang and Wang (2022). This scale comprehensively reflects an individual’s anxiety or worry when interacting with AI, comprising four dimensions: learning, job replacement, sociotechnical blindness, and AI configuration (Cronbach’s *α* = 0.874). A sample item is, “Learning to understand all of the special functions associated with an AI technique/product makes me anxious.”

Entrepreneurial resilience was measured using the 4-item scale from Santoro et al. (2020), which is suitable for assessing entrepreneurs’ coping abilities when facing external risks. (Cronbach’s *α* = 0.899) A sample item includes, “I actively look for ways to replace the losses I encounter in life.”

Social support was adapted from Xiao (1994) and L. Liu et al. (2025). It comprises three dimensions: objective support (3 items), subjective support (4 items), and support utilization (3 items). A higher total score indicates a higher level of social support.

Potential confounding factors were accounted for by incorporating a set of control variables in the analysis. At the individual level, these comprised the entrepreneurs’ gender, age, and education level. At the firm level, controls included the firm age, size and industry.

### 3.3. Statistical Analyses

This research proposes and tests a conceptual framework that examines how AI literacy influences entrepreneurial resilience, by incorporating AI anxiety as a mediator and social support as a moderating influence. Structural Equation Modeling (SEM) is capable of simultaneously examining complex relationships among multiple variables and effectively revealing the structural relationships between latent variables. Therefore, SEM was selected as the analytical method for this study. First, the reliability and validity of the constructs were examined. The internal consistency of the scales was evaluated using Cronbach’s *α*. Convergent and discriminant validity were assessed by examining standardized factor loadings, composite reliability (CR), and average variance extracted (AVE). Having established a reliable and valid measurement model, we constructed a structural equation model to examine the hypothesised path relationships. To evaluate the overall model fit, multiple indices were examined, including χ^2^/df, RMSEA, CFI, and TLI. Initial analyses, including descriptive statistics, correlations, and reliability assessments, were performed using SPSS 27.0. Confirmatory factor analysis and hypothesis testing were then conducted in Mplus 8.3.

## 4. Results

### 4.1. Descriptive Statistics

As shown in Table 2, we conducted descriptive statistics and correlation analysis. AI literacy was significantly negatively correlated with AI anxiety (r = −0.402, *p* < 0.001) and significantly positively correlated with entrepreneurial resilience (r = 0.386, *p* < 0.001). Additionally, AI anxiety was significantly negatively correlated with entrepreneurial resilience (r = −0.475, *p* < 0.001) and all three dimensions of social support. The data also showed that objective support, subjective support, and utilization of support all had significant positive correlations with entrepreneurial resilience. The correlations among the main study variables provide preliminary support for the proposed relationships.

### 4.2. Reliability and Validity

We implemented several measures to mitigate CMB. During the data collection phase, we distributed anonymous questionnaires and assured participants of confidentiality, which eliminated the need for identifiable information. We also conducted two waves of data collection. Statistically, Harman’s single-factor test was conducted. The results showed that the first principal component had an eigenvalue greater than 1, explaining 15.43% of the variance, which did not exceed 40%. These results indicate that CMB is not a concern in this study.

We further assessed reliability and validity, with results presented in Table 3. The Cronbach’s α coefficients for all variables were above 0.85, confirming high reliability. All standardized factor loadings for the items exceeded 0.7. Furthermore, the AVE for each variable was above 0.5, and the CR ranged from 0.874 to 0.914. These results indicate that the internal reliability, convergent validity, and discriminant validity all reached ideal levels.

### 4.3. Hypothesis Testing

**Figure 2:**
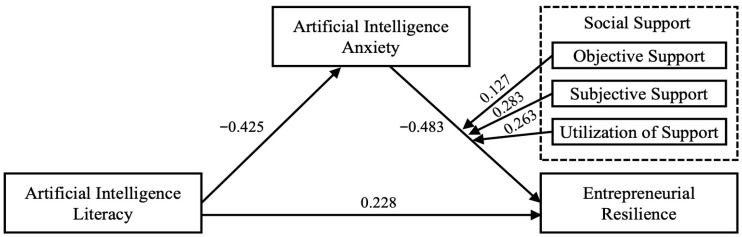
Result of hypothesis test.

This study tested the proposed hypotheses while accounting for all relevant control variables. The structural equation model established in this study demonstrated a good fit to the data (χ^2^/df = 1.656, CFI = 0.949, TLI = 0.943, RMSEA = 0.041). The standardized path coefficients and the analysis of direct and indirect effects are reported in Figure 2 and Table 4, respectively. The analysis revealed a direct, positive, and significant effect of AI literacy on entrepreneurial resilience (*β* = 0.228, *p* < 0.001), suggesting that without introducing any mediating or moderating factors, entrepreneurs’ AI literacy can directly enhance their entrepreneurial resilience. Furthermore, AI literacy was negatively correlated with the mediator, AI anxiety (β = −0.425, *p* < 0.001). Thus, higher levels of AI literacy predict a decrease in AI anxiety, providing support for H2. We also found that AI anxiety significantly and negatively impacted entrepreneurial resilience (*β* = −0.483, *p* < 0.001), supporting H3. We further performed a bootstrap analysis (5000 samples) to rigorously test the indirect effect and verify mediation. The analysis confirmed a significant indirect effect of AI literacy on entrepreneurial resilience through AI anxiety (Effect = 0.205, 95% CI [0.126, 0.299]). Moreover, the results showed that AI literacy explained 38.7% of the variance in AI anxiety (R^2^ = 0.387), while AI literacy and AI anxiety together explained 49.3% of the variance in entrepreneurial resilience (R^2^ = 0.493). Thus, AI anxiety serves as a mediator between AI literacy and resilience, supporting H4.

### 4.4. Additional Analysis

Table 5 presents the results of the moderation analysis. While all three types of social support significantly mitigated the negative effect of AI anxiety on entrepreneurial resilience, the strength of their buffering effects differed. The moderating effect was strongest for subjective support (*β* = 0.283, *p* < 0.001). Simple slope analysis demonstrated that high subjective support (+1 SD) significantly buffered the negative impact of AI anxiety on entrepreneurial resilience (−0.139), compared to the low support condition (−0.765), thereby supporting H5b. Likewise, utilization of support demonstrated a significant moderating effect (*β* = 0.263, *p* < 0.001). In the low support utilization group, the simple slope was −0.689, whereas in the high support utilization group, the slope’s absolute value significantly decreased (−0.209). This indicates that actively seeking and utilizing support resources can attenuate the negative impact of AI anxiety on entrepreneurial resilience, supporting H5c. By contrast, while still statistically significant, the moderating effect of objective support was notably weaker than that of subjective support and support utilization (*β* = 0.127, *p* < 0.05). Simple slope analysis further underscored this effect. Under high objective support, the negative impact of AI anxiety was only slightly reduced (–0.324), relative to the low support condition (–0.572). We will discuss this phenomenon further in the following section.

## 5. Discussion

First, this study reveals the positive impact of entrepreneurs’ AI literacy on their entrepreneurial resilience. It extends the theoretical scope and application of AI literacy from its primary focus on the educational domain (Imjai et al., 2025; Wijaya et al., 2024; Shi et al., 2025) to the entrepreneurial context, establishing AI literacy as an essential ability for surviving and thriving in AI entrepreneurial era. Furthermore, the study shifts the research focus from the predictive role of AI literacy on entrepreneurial intention (Pham et al., 2025; Duong, 2025; Nguyen et al., 2024), to its effect on enhancing resilience in the actual entrepreneurial process. This finding aligns with the established conclusion that technological literacy can foster entrepreneurial resilience (Shatila et al., 2025; Santos et al., 2023) and provides empirical evidence from the AI context.

Second, the results provide empirical support for the significant associations linking AI literacy, AI anxiety, and entrepreneurial resilience, indicating that AI literacy was found to positively affect entrepreneurial resilience via the mediating role of AI anxiety. Extending prior research in which AI anxiety has been approached primarily as an intervention target or predictor (Tarsuslu et al., 2025; Yao & Xi, 2025; Y. M. Wang et al., 2024; Y. Y. Wang & Wang, 2022), this study position it as a pivotal mediating mechanism. The observed significant negative correlation between AI literacy and AI anxiety is consistent with prior findings (Cengiz & Peker, 2025; Qiu et al., 2025; Pan et al., 2025), which further reinforcing the perspective that these two concepts should not be considered completely in isolation (Schiavo et al., 2024). Furthermore, this study reveals that lower levels of AI anxiety are significantly associated with higher entrepreneurial resilience. This suggests that when AI anxiety is alleviated, entrepreneurs experience a reduction in cognitive load and clearer judgment, thereby enhancing their ability to cope with adversity. These findings deepen our understanding of the internal mechanism through which technological literacy influences resilience via emotion regulation, confirming the critical role of managing technology related anxiety in the AI-driven entrepreneurial context.

Finally, this study moves beyond the assumption of social support’s general effectiveness by revealing the differential impacts of its three forms in buffering the influence of AI anxiety on entrepreneurial resilience. This finding aligns with the view that different types of social support play distinct roles (L. Liu et al., 2025; Huang et al., 2024) and provides concrete evidence for their divergent functions within the moderating mechanism linking anxiety to resilience. Empirically, the moderating effects of subjective support and support utilization were significantly stronger than those of objective support. This finding suggests that when coping with psychological threats induced by technological change, emotional reassurance and individual agency are far more impactful than the mere provision of tangible or informational aid.

It is important to clarify that the relatively weak moderating effect of objective social support does not imply a lack of value. Objective resources, such as funding, equipment, and information, play a fundamental role in sustaining entrepreneurial activities and ability development. Therefore, it is necessary to further explore the reasons for its weak moderating role in this study. One possible explanation is a mismatch between the supply logic of objective resources and the demand logic of entrepreneurs in the AI anxiety context. AI anxiety is essentially not just a technical problem but rather a psychological stress reaction to a crisis of value, control, and meaning. Subjective support directly addresses emotional needs, while the utilization of support precisely facilitates the mobilization of resources. In contrast, objective support is more applicable to solving known technical challenges, and its pathway to alleviating diffuse, endogenously generated anxiety is relatively indirect. Furthermore, objective support may involve usage or cognitive barriers, and its effectiveness requires entrepreneurs to possess corresponding competencies.

## 6. Conclusions

### 6.1. Theoretical Implications

This study contributes to the literature by advancing understanding in three primary areas. First, existing literature has predominantly situated AI literacy within educational contexts (Imjai et al., 2025; Wijaya et al., 2024; Shi et al., 2025). Even within the field of entrepreneurship research, investigations have been largely limited to its role in predicting entrepreneurial intention (Pham et al., 2025; Duong, 2025; Nguyen et al., 2024). This lacks in-depth exploration of the practical value of AI literacy among entrepreneurs in real business environments. By empirically examining its mechanism in enhancing entrepreneurial resilience, this research not only shifts the context from educational settings to dynamic, high-pressure entrepreneurial practice but also accomplishes a theoretical expansion from intention prediction to resilience enhancement. Furthermore, this study advances the literature by shifting the scholarly focus from digital literacy to AI literacy (Santos et al., 2023; Shatila et al., 2025), a pivotal evolution in technological competencies. By establishing AI literacy as a critical ability for enhancing entrepreneurial resilience, this exploration enriches the research scope and offers a new perspective for understanding the function of advanced technological abilities in contexts of entrepreneurial stress.

Second, by applying COR theory to the AI entrepreneurial context, our study reveals the internal mechanism through which entrepreneurs leverage AI literacy to enhance their resilience. Within this theoretical framework, AI literacy is conceptualized as a critical personal resource, while AI anxiety is identified as a persistent psychological stressor and resource threat. The findings indicate that enhancing entrepreneurial resilience requires not only acquiring technical ability (AI literacy) but also effectively coping with stressful contexts and perceived resource threats (AI anxiety). This resilience enhancement model resonates with the dialectical wisdom of “forewarned is forearmed” in traditional Chinese thought. These findings point to the importance of psychological resource management in AI entrepreneurship. This suggests that alongside enhancing their technical AI capabilities, entrepreneurs should also identify and regulate anxiety to prevent resource depletion and interference, thereby strengthening resilience. This finding offers fruitful new avenues for the application of COR theory, particularly at the interdisciplinary nexus of entrepreneurship research, technology adoption, and psychology.

Third, this study elucidates the critical boundary conditions under which AI anxiety affects entrepreneurial resilience by examining the moderating role of social support. The findings illustrate how social support embodies the resource caravans principle of COR theory, providing a pathway for entrepreneurs to conserve psychological resources under stress and subsequently strengthen their resilience. The absence of such support is likely to result in both reduced coping efficacy and increased psychological fragility among entrepreneurs. Particularly, we found that the moderating effect of objective support was significantly weaker compared to that of subjective support and support utilization. This indicates that the effectiveness of resource channels is highly context-dependent, with their value being contingent on a precise match to the individual’s needs and specific pressure types. By examining the synergistic interaction between internal and external resources under complex stress, this study extends the application of COR theory in entrepreneurship and provides a theoretical foundation for exploring resource-stress matching.

### 6.2. Managerial Implications

First, entrepreneurs should systematically develop AI literacy as a critical entrepreneurial ability. To achieve this, they need to proactively acquire AI knowledge to make rational technology adoption decisions. Beyond that, they should continuously improve their practical technical abilities through scenario-based applications and focus on building a human–machine collaborative decision-making mechanism. Equally critical is the need to establish a prudent and responsible AI ethics framework, ensuring that technological applications adhere to ethical standards and social responsibility. Accordingly, we recommend that entrepreneurs engage in learning activities such as specialized AI entrepreneurship workshops and case studies in industry salons to gradually strengthen their mastery of the technological environment through the integration of knowledge and practice.

Second, entrepreneurs must incorporate the identification and intervention of AI anxiety into their entrepreneurial risk management systems. Our study identifies AI anxiety as a critical psychological mechanism through which AI literacy influences entrepreneurial resilience, highlighting the necessity of managing this anxiety. Entrepreneurs can implement routine emotional self-assessments using tools such as anxiety scales. This practice facilitates the early detection of signals associated with resource depletion. If excessive worry is detected, entrepreneurs should view it as a common reaction during the technology adaptation phase and actively seek to mitigate it through methods such as discussion and sharing, psychological counseling, or mindfulness practices. Furthermore, entrepreneurial support organizations can regularly organize relevant workshops or technology roundtables. This can help create a safe and open atmosphere, enabling entrepreneurs to better manage and alleviate their psychological stress.

Finally, in the high-pressure entrepreneurial environment characterized by the rapid iteration of AI technology, entrepreneurs must strategically integrate and leverage social support resources. Specifically, they should not view the support system as a monolithic or passive aid. Instead, it should be treated as a dynamically configurable resource system. Entrepreneurs should intentionally cultivate a core relational network capable of providing emotional resonance and value affirmation. Even more critical is fostering the initiative to actively seek and utilize resources. Entrepreneurs can do this, for instance, by joining AI technology communities or seeking expert guidance. Moreover, emphasis should be placed on enhancing the utilization efficiency of objective support resources, such as open industry AI big datasets, to avoid the predicament of having resources but being unable to use them effectively. Last but not least, support platforms should shift from a logic of unilateral resource provision to creating precision-matching mechanisms that align resources with entrepreneurs’ actual needs.

### 6.3. Limitation and Future Research

First, while the time-lagged design used in this study advances causal inference, it does not equate to a fully longitudinal approach. Hence, subsequent studies should employ a multi-wave longitudinal tracking design or diary method to more precisely capture their trajectories over time and their dynamic interaction mechanisms.

Second, this study operationalizes AI literacy as an integrated construct, offering a clearer theoretical model for verifying its role as a critical resource in AI era. However, it does not reveal the potential differential impact mechanisms of its distinct dimensions (e.g., AI Basics, Proficiency, Insight, Analysis, and Ethics). Future research should therefore investigate the unique pathways through which each sub-dimension influences entrepreneurial outcomes, thereby building a more nuanced understanding of this critical literacy.

Third, the mechanism through which AI literacy affects entrepreneurial resilience is complex. Beyond the pathways examined, other significant mediating or moderating factors may exist. At the individual level, factors such as AI self-efficacy, technological adaptability, and entrepreneurial experience could play a role. At the contextual level, external conditions such as industry characteristics, the level of AI policy support by country, and the pace of technological change may serve as crucial boundary conditions. Future research could integrate these factors into a comprehensive theoretical framework to further investigate the multiple pathways and boundary conditions of this relationship.

Finally, A valuable direction for future studies involves assessing the broader applicability of the framework through systematic cross-cultural, cross-industry, and cross-developmental-stage comparisons. For example, does the protective effect of AI literacy on resilience differ between entrepreneurs in high-tech industries and those in traditional industries? In countries or regions with varying levels of policy support for the AI industry, do entrepreneurs’ AI anxiety levels and their subsequent impact on resilience also differ? Such research would greatly enrich our understanding of the psychological and behavioral adaptations of entrepreneurs in the AI era.

## Figures and Tables

**Table 1:** Demographic information of participants (*N* = 330).

Variable	Classification	N	Proportion(%)
Gender	Male	230	69.7
	Female	100	30.3
Age	≤25	8	2.4
	26–35	140	42.4
	36–45	133	40.3
	≥46	49	14.9
Education	College diploma or below	57	17.3
	Bachelor’s degree	184	55.7
	Master’s degree or higher	89	27.0
Firm age	≤1	85	25.8
	2–4	177	53.6
	≥5	68	20.6
Firm size	≤10 employees	108	32.7
	11–50	147	44.6
	≥51	75	22.7
Industry	Software and electronic information technology	62	18.8
	Manufacturing	53	16.1
	Culture and media	38	11.5
	Retail	47	14.3
	Medical and health services	49	14.8
	Energy	40	12.1
	others	41	12.4

**Table 2:** Descriptive statistics and correlation analysis between variables (*N* = 330).

Variable	Mean	SD	1	2	3	4	5	6
1. Artificial Intelligence Literacy	3.408	1.016	1					
2. Artificial Intelligence Anxiety	3.099	1.024	−0.402 ***	1				
3. Entrepreneurial Resilience	3.501	0.979	0.386 ***	−0.475 ***	1			
4. Objective Support	3.215	0.982	0.183 *	−0.159 *	0.225 **	1		
5. Subjective Support	3.472	1.105	0.203 *	−0.352 ***	0.339 ***	0.418 ***	1	
6. Utilization of Support	3.482	0.913	0.247 **	−0.308 ***	0.346 ***	0.386 ***	0.524 ***	1

Notes: * *p* < 0.05; ** *p* < 0.01; *** *p* < 0.001.

**Table 3:** Measurement Model.

Variables	Items	Factor Loadings	CR	AVE
Artificial Intelligence Literacy	AIL1	0.832	0.914	0.697
	AIL2	0.854		
	AIL3	0.843		
	AIL4	0.821		
	AIL5	0.815		
	AIL6	0.824		
	AIL7	0.901		
	AIL8	0.853		
	AIL9	0.872		
	AIL10	0.816		
	AIL11	0.838		
Artificial Intelligence Anxiety	AIA1	0.823	0.874	0.624
	AIA2	0.841		
	AIA3	0.832		
	AIA4	0.847		
	AIA5	0.856		
	AIA6	0.810		
	AIA7	0.862		
	AIA8	0.855		
	AIA9	0.829		
	AIA10	0.842		
	AIA11	0.813		
	AIA12	0.839		
	AIA13	0.866		
	AIA14	0.857		
	AIA15	0.839		
	AIA16	0.824		
	AIA17	0.825		
	AIA18	0.822		
	AIA19	0.811		
	AIA20	0.838		
	AIA21	0.849		
Entrepreneurial Resilience	ER1	0.892	0.898	0.685
	ER2	0.885		
	ER3	0.863		
	ER4	0.904		
Objective Support	OS1	0.859	0.878	0.672
	OS2	0.822		
	OS3	0.860		
Subjective Support	SS1	0.876	0.905	0.702
	SS2	0.882		
	SS3	0.901		
	SS4	0.895		
Utilization of Support	UOS1	0.868	0.882	0.613
	UOS2	0.847		
	UOS3	0.871		

**Table 4:** Direct and indirect effects of each model path.

**Direct Effect**	**β**	**SE**
AI Literacy → Entrepreneurial Resilience	0.228 ***	0.066
AI Literacy → AI Anxiety	−0.425 ***	0.071
AI Anxiety → Entrepreneurial Resilience	−0.483 ***	0.063
**Indirect Effect**	**Estimate**	**95% CI**
AI Literacy → AI Anxiety→Entrepreneurial Resilience	0.205	[0.126, 0.299]

Note: *** *p* < 0.001. SE = standard error. CI = confidence interval.

**Table 5:** Result of moderation effect.

Main Variables	Entrepreneurial Resilience					
	Estimate	SE	Estimate	SE	Estimate	SE
Artificial Intelligence Anxiety (AIA)	−0.447 ***	0.050				
Objective Support (OS)	0.138 *	0.074				
AIA × OS	0.127 *	0.066				
Artificial Intelligence Anxiety (AIA)			−0.452 ***	0.051		
Subjective Support (SS)			0.352 ***	0.045		
AIA × SS			0.283 ***	0.055		
Artificial Intelligence Anxiety (AIA)					−0.449 ***	0.049
Utilization of Support (UOS)					0.341 ***	0.046
AIA × UOS					0.263 ***	0.054

Notes: * *p* < 0.05; *** *p* < 0.001.

## Data Availability

The raw data supporting the conclusions of this article will be made available by the authors, upon reasonable request.

## Abbreviations

The following abbreviations are used in this manuscript:

AI	Artificial intelligence
AIA	Artificial Intelligence Anxiety
AIL	Artificial Intelligence Literacy
BANI	Brittle, Anxious, Nonlinear, Incomprehensible
COR	Conservation of Resources
ER	Entrepreneurial Resilience
OS	Objective Support
SS	Subjective Support
UOS	Utilization of Support
